# Offense and Defense in Granulomatous Inflammation Disease

**DOI:** 10.3389/fcimb.2022.797749

**Published:** 2022-06-29

**Authors:** Xinwen Wang, Yuan Liu

**Affiliations:** ^1^Shaanxi Clinical Research Center for Oral Diseases, National Clinical Research Center for Oral Diseases, State Key Laboratory of Military Stomatology, Department of Oral Medicine, School of Stomatology, The Fourth Military Medical University, Xi’an, China; ^2^Shaanxi International Joint Research Center for Oral Diseases, State Key Laboratory of Military Stomatology, Department of Histology and Pathology, School of Stomatology, The Fourth Military Medical University, Xi’an, China

**Keywords:** bacteria, granulomatous inflammation diseases, predisposing factors, immune response, gene, intracellular bacteria

## Abstract

Granulomatous inflammation (GI) diseases are a group of chronic inflammation disorders characterized by focal collections of multinucleated giant cells, epithelioid cells and macrophages, with or without necrosis. GI diseases are closely related to microbes, especially virulent intracellular bacterial infections are important factors in the progression of these diseases. They employ a range of strategies to survive the stresses imposed upon them and persist in host cells, becoming the initiator of the fighting. Microbe-host communication is essential to maintain functions of a healthy host, so defense capacity of hosts is another influence factor, which is thought to combine to determine the result of the fighting. With the development of gene research technology, many human genetic loci were identified to be involved in GI diseases susceptibility, providing more insights into and knowledge about GI diseases. The current review aims to provide an update on the most recent progress in the identification and characterization of bacteria in GI diseases in a variety of organ systems and clinical conditions, and examine the invasion and escape mechanisms of pathogens that have been demonstrated in previous studies, we also review the existing data on the predictive factors of the host, mainly on genetic findings. These strategies may improve our understanding of the mechanisms underlying GI diseases, and open new avenues for the study of the associated conditions in the future.

## Introduction

Granulomatous inflammation (GI) is a special type of chronic inflammation, which is characterized by focal collections of multinucleated giant cells, epithelioid cells and macrophages in response to a persistent inflammatory stimulus (Williams and Williams, 1983). GI is a protective response to encapsulate foreign material, and is found throughout both vertebrate and invertebrate species. A wide range of stimuli can result in GI, including microbes, parasites and fungi (Shah et al., 2017), some viruses have also been described in association with GI diseases.

Among these stimuli, microbes are the important triggers for the development of GI. In recent decades, an increasing body of studies implicated microbes and their alterations in many GI diseases, i.e., tuberculosis (TB), leprae, Crohn’s disease (CD) and Buruli ulcer (BU). Evidence of *Mycobacterium, Nocardia, Salmonella*, etc., involved in GI pathogenesis has been found. In some GI diseases, clear proof-of-concept of causality is still lacking, but the possibility of an infectious origin has been postulated.

The complexity of GI pathogenesis, involving multiple distinct elements, pathogens maybe necessary but not always sufficient, especially for the GI diseases initiated by less virulent bacteria or resident bacteria, A previous study showed that a sibling of a CD patient who develops the diseases is at 13 to 36-fold higher risk compared with that of the general population (Ahmad et al., 2001), therefore the genetic factors of hosts have long been considered another major contributor to GIs. With widely applied genome-wide approaches, many genetic loci have been identified to be involved in GI disease susceptibility, moreover some unexpected overlap in genetic architecture between different GI diseases have been revealed, which further emphasizes the genetic role in the pathogenesis of GI diseases, although other predisposing conditions of the host, (including diet and healthy status), and environmental risk factors may also play a role (Alcais et al., 2009; Fox et al., 2016).

Here, we will focus on the bacterial involvement in GIs, parasites, fungi, virus and non-infectious agents will not be discussed, and provide an overview of the recent advances in both bacteriology and genetics that impact the development of GI diseases.

## Microbes Associated With Granulomatous Inflammation

### Actinobacteria

The members of *Actinobacteria* are by far the most common causes of GI diseases (**Table 1**). TB, caused by infection with *Mycobacterium tuberculosis* is a classic and ancient GI disease, and remains one of the leading causes of morbidity and mortality globally (Lawn and Zumla, 2011). Leprosy is a GI disease caused by *M. leprae*, another member of *Mycobacterium* spp. that mainly affects skin and peripheral nerves (Kaplan and Cohn, 1986). Other GI diseases verified by scientific research as having an association with *Mycobacterium* spp. include BU (Mac et al., 1948; Ruf et al., 2011), a chronic necrotizing GI skin disease; sarcoidosis, a multisystem GI diseases (Esteves et al., 2016); CD, a GI disease of the gastrointestinal tract (Kirkwood et al., 2009); malakoplakia, a rare GI disease that affects a wide variety of organs (Remond et al., 1994; Mitchell and Dugas, 2019); and Takayasu arteritis, a rare large-vessel GI vasculitis (Mwipatayi et al., 2005; Soto et al., 2012).

**Table 1:** Overview of the bacterial phylotypes involved in GI diseases and their biological characteristics.

Bacterial phylotypes		Biological characteristics	Related GI diseases	References
***Actinobacteria***	*Mycobacterium tuberculosis*	IB	TB, Takayasu arteritis, Sarcoidosis, CGD	(Lawn and Zumla, 2011; Soto et al., 2012; Conti et al., 2016; Lee et al., 2019)
	*Mycobacterium leprae*	IB	Leprae	(Kaplan and Cohn, 1986)
	*Mycobacterium ulcerans*	IB	BU	(Mac et al., 1948)
	*Mycobacterium paratuberculosis*	IB	CD	(Kirkwood et al., 2009; Sibartie et al., 2010)
	*Mycobacterium simiae*	IB	Malakoplakia	(Chitasombat and Wattanatranon, 2015)
	*Propionibacterium acnes*	N/A	Sarcoidosis	(Abe et al., 1984; Robinson et al., 2013)
	*Actinomyces israelii*	N/A	Actinomycosis	(Figdor and Davies, 1997)
	*Nocardia*	IB	CGD, Nocardiosis	(Dorman et al., 2002; Betran et al., 2009)
***Proteobacteria***	*Escherichia coli*	IB	CD, Malakoplakia	(Stanton and Maxted, 1981; Freeman, 2014)
	*Helicobacter pylori*	IB	CD	(Fallone and Bitton, 2008)
	*Campylobacter concisus*	IB	CD	(Mahendran et al., 2013; Zhang et al., 2014)
	*Burkholderia*	IB	CGD	(Vining et al., 2014)
	*Chromobacterium*	N/A	CGD	(Sureisen et al., 2008)
	*Serratia marcescens*	N/A	CGD	(Barbato et al., 2014)
	*Brucella*	IB	Brucellosis	(Olsen and Palmer, 2014)
	*Coxiella burnetii*	IB	Granulomatous hepatitis	(Aguilar-Olivos et al., 2013)
	*Francisella tularensis*	IB	Tularemia	(Lamps et al., 2004)
	*Yersinia*	IB	Granulomatous appendicitis	(Pal, 2014)
	*Salmonella typhi*	IB	Bone marrow granuloma	(Muniraj et al., 2015)
	*Bartonella henselae*	IB	Cat scratch	(Scott et al., 1996)
	*Klebsiella*	N/A	Scleroma	(Sedano et al., 1996)
	*Rickettsia*	IB	Granulomatous conjunctivitis	(Abroug et al., 2015)
	*Proteus*	N/A	Botryomycosis	(Bonifaz and Carrasco, 1996)
***Firmicutes***	*Staphylococcus aureus*	IB	CGD, GPA, Malakoplakia	(Remond et al., 1994; Zycinska et al., 2008; Laudien et al., 2010)
	*Streptococcus*	N/A	CD, OFG	(Liu et al., 1995; Goel et al., 2019)
	*Clostridium difficile*	N/A	CD	(Rodemann et al., 2007)
	*Listeria monocytogenes*	IB	CD, Granulomatous hepatitis	(Gebauer et al., 1989; Liu et al., 1995)
***Spirochaetes***	*Borrelia burgdorferi*	N/A	Sarcoidosis, OFG	(Jacob, 1989; Liu, 1993)
***Chlamydiae***	*Chlamydia*	IB	Granulomatous Peritonitis	(Tondo-Steele et al., 2020)
***Tenericutes***	*Mycoplasma pneumoniae*	IB	CD	(Chen et al., 2001)

IB, intracellular bacteria; N/A, not intracellular bacteria or not sure; TB, tuberculosis; CD, Crohn’s disease; BU, Buruli ulcer; CGD, chronic granulomatous disease; GPA, Wegener’s granulomatosis; OFG, orofacial granulomatosis.Survival and Persistence of Pathogens.

*Mycobacterium* spp. are aerobic rod-shaped, obligate intracellular bacteria enriched in lipids with long-chain mycolic acids in cell envelope (Daffe and Etienne, 1999; Rahlwes et al., 2019), and there are many common biological characteristics among them, therefore, scientists presumed that they may have evolved from a common ancestor (Mostowy and Behr, 2005; Stinear et al., 2007; Roltgen et al., 2012; Boritsch et al., 2014). *Mycobacterium* spp. can exert pathogenic capacity in a direct way, for example 6-kDa early secreted antigenic target (ESAT-6) secretion system 1 (ESX-1), possess membrane-lysing activity, and induce host cell necrosis and spread of bacteria to adjacent cells (Van Der Wel et al., 2007; Smith et al., 2008; Welin et al., 2011); Mycolic acid, hallmarks of *Mycobacterium* spp. serves as a special barrier critical for many of the disease-inducing and physiological aspects of *Mycobacterium* (Forrellad et al., 2013). Also, *Mycobacterium* spp. can exert pathogenic capacity in an indirect way, e.g. they release a mannose-containing glycoconjugate, which can impair the ability of macrophages to kill other phagocytosed pathogens (Mpofu et al., 2007; Friswell et al., 2010), the extensive sequence homology between human stress proteins and *Mycobacterium* may cause cross-reactivity against vascular peptides that mimic the antigens of *M. tuberculosis*, which might be the etiological factor of Takayasu arteritis (Castillo-Martinez and Amezcua-Guerra, 2012).

In addition to *Mycobacterium* spp., other numbers of *Actinobacteria*, such as *Propionibacterium*, *Actinomyces* and *Nocardia* have been also reported in GI diseases (**Table 1**). More specific strategies they employ will be discussed below.

### Proteobacteria

*Escherichia coli (E. coli)* is the most typical and the best-characterized species of the *Proteobacteria* phylum in GI, mainly in CD. In contrast to pathogenic *Mycobacterium* species, *E. coli* is a resident bacterium in normal intestinal flora, where it plays an important role in maintaining normal intestinal homeostasis. However, over the years, many studies have implicated *E. coli* as a provocative factor for the development of CD (Freeman, 2014), although a direct causal relationship between *E.coli* and CD has not been established in humans. *E.coli* is also a commonly cultured microorganism alone or along with other pathogens in malakoplakia lesions (Stanton and Maxted, 1981).

*E. coli* usually colonizes different epithelial surfaces with genetic and phenotypic diversities. However, upon infection *E.coli* from the outer loose mucin layer can penetrate and interact with the epithelial lining and deeper layers, showing unique adherent and invasive properties, so that it was named adherent invasive *E. coli* (AIEC). Studies showed that AIEC adhere to intestinal epithelial cells through carcinoembryonic antigen-related cell adhesion molecule 6 receptors (CEACAM6 receptors), enter cells *via* a macropinocytosis-like process, lyse the endocytic vacuole (Boudeau et al., 1999), survive and replicate within both epithelial cells and macrophages (Mann and Saeed, 2012), which may promote granuloma formation (Glasser et al., 2001; Barnich et al., 2007; Carvalho et al., 2009; Matricon et al., 2010).

In addition to *E. coli*, there are other *Proteobacteria* species that have been reported to be associated with GI diseases, including *Gammaproteobacteria* members *Francisella, Coxiella, Yersinia, Salmonella, Proteus* and *Serratia*; *Alphaproteobacteria* members, *Brucella, Bartonella*, and *Rickettsia*; *Betaproteobacteria* members, *Burkholderia*, *Klebsiella* and *chromobacterium; Epsilonproteobacteria* members, *Helicobacter* and *Campylobacter* (**Table 1**).

### Firmicutes

Of the phylotypes of the *Firmicutes* phylum, *Staphylococcus aureus*, *Streptococcus, Clostridium* and *Listeria* were reported to be involved in GI diseases (**Table 1**). They are all Gram-positive pathogens, and widely distributed in nature. *S. aureus* infection is the signature complication of chronic granulomatous disease (CGD), which is a genetic immune disease caused by the deficiency of the phagocyte transmembrane nicotinamide adenine dinucleotide (NADPH) oxidase (Buvelot et al., 2017). *S. aureus* was also occasionally isolated from some malakoplakia patients (Remond et al., 1994). In search of a specific pathogenic bacterial agent for Wegener’s granulomatosis (also known as granulomatosis with polyangiitis, GPA), it was found that chronic nasal carriage of *S. aureus* is approximately three times higher in GPA patients than in healthy control (Stegeman et al., 1994).

*Streptococcus* and *Listeria* were identified immunochemically in giant cells, macrophages and lymph nodes of CD patients (Liu et al., 1995). In a recent study of orofacial granulomatosis (OFG), which is considered to be close related to CD, the increased abundance of the *Streptococcus* was found in saliva of OFG patients (Goel et al., 2019).

Despite clinical evidence suggesting that these members of *Firmicutes* may be implicated in the pathophysiology of GI diseases, laboratory investigation of the possible mechanisms by which they are involved in GI is relatively limited. As Streptococcal immunoreactivity was also found in some normal tissue, Liu et al. concluded that *Streptococcus* more than likely acted as secondary rather than primary agents associated with CD (Liu et al., 1995). However, by virtue of that they possess the ability to produce putative virulence determinants, evade from phagocytes killing, they as mediators of GI are still attractive targets for further investigation.

### Spirochaetes

*Spirochaetes*, a group of gram-negative bacteria, have long and spiral cell bodies, endoflagella that reside in the periplasmic space and flagella-dependent motility that sets them apart from other bacteria (Johnson, 1977; Harwood and Canale-Parola, 1984). Taxonomically, the phylum *Spirochaetes* is classified into the *Spirochaetaceae*, *Leptospiraceae, Brevinemataceae* and *Brachyspiraceae* families. They are very heterogeneous, with cell dimensions varying from diameters of 0.09μm to lengths of 500μm. The morphologies of the cell body and endoflagella greatly differ among species. Compared with that of other bacteria, the scientific understating of the physiology and molecular biology of spirochetes remains very limited. Flagella and motility are known virulence factors of pathogenic *Spirochaetes*, and are related to invasion and adhesion (Josenhans and Suerbaum, 2002; Haiko and Westerlund-Wikstrom, 2013).

The major means of spirochetes motility is swimming. They are attracted to areas of higher viscosity and exhibit a viscosity-dependent increase in swimming speed (Petrino and Doetsch, 1978), which may assist the accumulation of *Spirochaetes* in the mucus layer *in vivo*. In addition, some *Spirochaetes* species can move on a solid surface through twitching motility (Wall and Kaiser, 1999). The spirochaetal movements over host cell surfaces have been shown to be related to the severity of the symptoms caused by this microorganism.

The hypothesis that *Spirochaetes* is a possible pathogen for sarcoidosis was first documented in 1989 in epidemiological studies (Jacob, 1989), but was not substantiated further (Arcangeli et al., 1994). There are also a few studies on the role of *Spirochaetes* in the etiology of OFG. The detection of antibodies against *Spirochaetes* in as many as 77.8% of patients with OFG has been reported (Liu, 1993; Liu et al., 1994). However, in some other studies, *Spirochaetes* were not detected (Muellegger et al., 2000), so currently, a definitive relationship between *Spirochaetes* and the pathogenesis of GI diseases is still debated, more studies are needed to illuminate the possible association.

In addition to the above pathogens, *Chlamydiae* and *Tenericutes* may be involved in GIs, and listed in **Table 1**.

**Figure 1:**
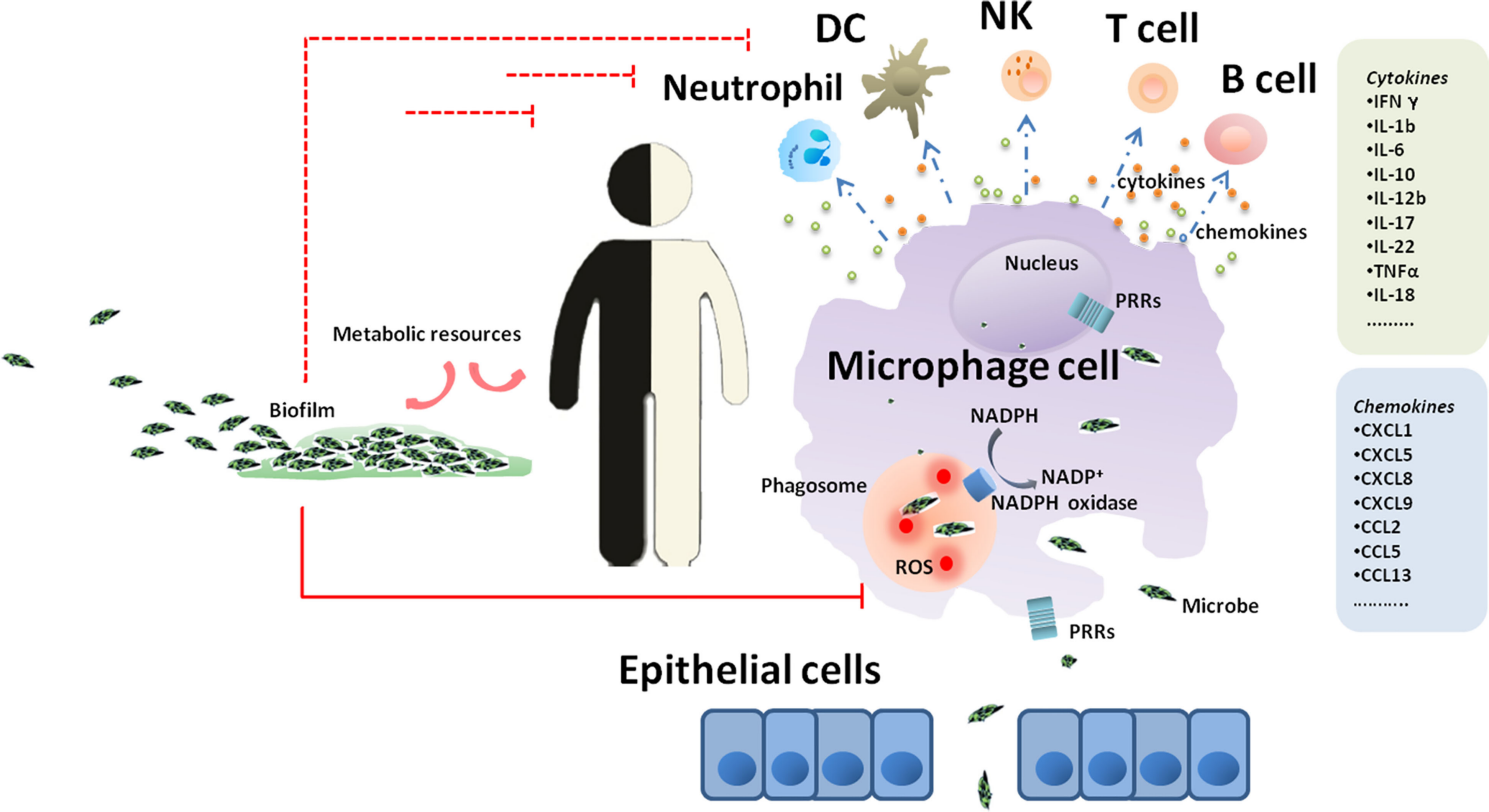
A journey of GI microbes in the host. Epithelium is the gateway for the introduction of GI microbes inside the host and subsequent progression of GI diseases. Once microbes get through the gateway, the process of phagocytosis is initiated, and macrophages are the first line of defense. In macrophages, invading microbes are engulfed by phagocytosis into phagosomal structures and encounter a complex and dynamic range of host defenses. The recognition of bacterial antigens by a range of pattern recognition receptors expressed by macrophages triggers a signaling cascade leading to the recruitment of a diverse cell type complement including neutrophils, dendritic cells, natural killer cells, T lymphocytes and B lymphocytes.

In the pathogenic bacteria known to be associated with GI diseases, the vast majority are intracellular bacteria (e.g., *M. tuberculosis, M. leprae, L. monocytogenes* and *S. typhimurium*) (**Table 1**). *E. coli* and *S. aureus* were considered extracellular pathogens for many years. However, recent growing evidence has demonstrated that they have the potential to invade and persist within host cells (Foster et al., 2014), including long-lived phagocytes (DCs and macrophages), as well as nonprofessional phagocytes (e.g., endothelial and epithelial cells, osteoblasts, and fibroblasts (Sinha et al., 1999). Compared to extracellular bacteria, intracellular pathogens are adapted to life within phagocytes, and employ a range of strategies to create a niche within host cells and survive the stresses imposed upon them, playing an underappreciated role in GI development (**Figure 1**). Various mechanisms by which GI pathogens survive and persist in host cells are discussed below.

### Adherence and Biofilm Formation

The capacity of long-term colonization of cell or tissue surfaces is the key characteristic of pathogens to cause infection. To initiate infection, the pathogens adhere to the host cells or tissues, which confer on them the potential to further invade host cells (Boudeau et al., 1999). For instance, the adhesions present in *Mycobacterium* spp. surface not only mediate the attachment of bacteria to the surfaces of tissue, but also are capable of sustaining their survival (De Lima et al., 2009; Silva et al., 2013); *S. aureus* cells express binding proteins that promote attachment to host proteins (e.g., fibronectin and laminin), and damaged tissue where the underlying layers exposed (Bisognano et al., 2004).

Once the bacteria attach to the surface of the cell or tissue, they initiate the synthesis of the extracellular matrix, which is composed of glycopeptides and other molecules, and then form fully developed biofilms (Zambrano and Kolter, 2005), unless some intracellular bacteria invade the host cells without the need of creating a biofilm. The biofilms provide a permeability barrier and stable community for the pathogens, host immune defenses are often inefficacious against bacteria growing in it (Costerton et al., 1999). The protection offered by biofilms makes a large proportion of bacterial biomass possible, which is another pathogenic trait. Furthermore, the high cell density in biofilms facilitates horizontal gene transfer between bacteria (Sorensen et al., 2005), playing a vital role in resistance to host defenses.

### Evasion of Host Immunity

The encounter between the host and pathogens leads to a multifaceted and complex immune response. The different cell lines of innate and adaptive immunity come into play at different times in the battle, composing an immune surveillance network that restricts infection. However, GI pathogens have a vast arsenal of defenses against the host immune system, and can evade it on many levels.

#### Escaping Immune surveillance

As the first line of defense during infections, macrophages are not only the primary targets of intracellular bacteria to be abused, but also the center of infection, which play decisive roles in host responses to intracellular bacteria through a myriad of defense strategies (**Figure 1**). The recognition of bacterial antigens by a range of pattern recognition receptors (PRRs) expressed by macrophages triggers a signaling cascade leading to the expression of cytokines, chemokines and peptides and to the activation and recruitment of a diverse cell type complement including neutrophils, natural killer (NK) cells and dendritic cells (DCs) (Ehlers and Schaible, 2012). PRR activation also initiates the expression of costimulatory molecules on the surface of macrophages and DCs, which is important for the onset of adaptive immunity (Nunes-Alves et al., 2014).

GI pathogens escape the immune surveillance in different ways. First, the pathogens subvert PRR recognition, and consequently avoid PRR activation (Baxt et al., 2013). For instance, *E. coli* invade bladder epithelial cells in a type 1 pilus-dependent way, thus shunning TLR4-mediated exocytic processes (Anderson et al., 2004). Second, the pathogens express some proteins that directly or indirectly impair immune reactions or even cause host cell death, thereby facilitating pathogen survival (Derrick and Morris, 2007). For instance, *S. aureus* expresses staphylococcal complement inhibitor, SCIN, which blocks opsonization by C3b and prevents C3a activation, which is important for mast cell degranulation (Rooijakkers et al., 2009). More recently, increasing attention has been directed at the emerging evidence showing that the host ubiquitin system is targeted by GI pathogens for immune evasion (Penn et al., 2018). Third, bacterial effectors were found to enter the nucleus of infected cell to destroy host nuclear processes, disturbing in host gene transcription and DNA replication or repair (Bierne and Cossart, 2012; Sharma et al., 2015).

#### Anti-Phagocytosis

After invasion, the pathogens are engulfed by phagocytosis into phagosomal structures that will fuse with endosomes, eventually with lysosomes, where the engulfed bacteria are destroyed (Turner et al., 2017). Intracellular bacteria can survive and acquire antiphagocytic capabilities. For instance, *M. tuberculosis* has an antiphagocytic capsule, which can limit and control the interaction of the bacterium with macrophages (Stokes et al., 2004); capsular polysaccharide (CP) and protein A (SpA) expressed by *S. aureus* (Thakker et al., 1998), stringent starvation protein A and the macrophage growth locus protein MglA secreted by *F. tularensis* (Bell et al., 2010; Bent et al., 2013), acid-tolerant proteins in *Mycobacteria* can help them to avoid phagocytic killing (Sundaramurthy et al., 2017); *M. tuberculosis* can even reduce the formation of phagosomes through increasing the expression of miR-142-3p and decreasing the actin binding protein N-Wasp in macrophages (Bettencourt et al., 2013); additionally, type VII secretion systems expressed in *Mycobacterium* spp. and alpha toxin expressed by *S. aureus* can help them to evade from phagocytes and subvert host defenses (Van Der Wel et al., 2007; Pang et al., 2010).

Autophagy is an essential process in which cytoplasmic constituents are engulfed by double-membrane autophagosomes, and finally degraded in vacuoles or lysosomes (Nakamura and Yoshimori, 2017). Autophagy was initially identified as a nonselective degradation to maintain cellular homeostasis. Recent studies have clearly shown that autophagy is also involved in the host defense against intracellular pathogen infection (Yoshimori and Amano, 2009; Manzanillo et al., 2013). In the phagocytes, intracellular pathogen is ubiquitinated and recognized by autophagy receptors, then is trapped in autophagosomes. Improving autophagy has been shown to promote bacterial killing (Gutierrez et al., 2004; Wang et al., 2013). Reversely, the autophagy process can be modulated by intracellular bacteria. *M. tuberculosis* and *M. Leprae* were all shown to dampen autophagy in human cells as an immune escape mechanism (Silva et al., 2017; Siregar et al., 2022). Similarly, infection with AIEC reduces the autophagy response in host cells by reducing the expression of proteins required for autophagy, whereby pathogens acquire enhanced intracellular survival (Palmela et al., 2018). *S. aureus* can escape autophagic degradation by blocking autophagy flux, inhibit the fusion of autophagosomes with lysosomes and increasing the pH in autolysosomes, also can utilize autophagy for its own intracellular survivial (Cai et al., 2020).

#### Anti-Oxidation

The pathogens engulfed by phagocytosis encounter a dynamic range of host defenses, including reactive oxygen, acidification, nitrogen intermediates, antimicrobial peptides (Stallings and Glickman, 2010) and environmental stringencies (Houben et al., 2012), of which the process known as an oxidative burst is crucial for the clearance of pathogens (Gan et al., 2008; Silva, 2010). Reactive oxygen species (ROS), which result from the activation of NADPH oxidase and generate ${\text{O}}_{2}^{-}$ , are highly toxic to bacteria. ROS production can initiate many oxidative reactions, either directly destroying protein, DNA, and lipids or indirectly damaging nucleic acids through oxidation of the nucleotide pool (Van Acker and Coenye, 2017). In addition, ROS can trigger inflammatory signaling cascades *via* genomic expression of proinflammatory regulators, transcription factors and protein kinase pathways, leading to an overactivated immune system (Choudhury and Macnee, 2017).

GI associated pathogens generally exert strong antioxidant activity. As the leading pathogen in GI diseases, *M. tuberculosis* has developed complex mechanisms to survive high oxygen stress (OS) burden in the host. Not only do the mycolic acids form a physical barrier to counter host-generated exogenous OS (Portevin et al., 2014), but they also secret a specific protein, namely, enhanced intracellular survivial (Eis) protein, which can sense ROS and respond in a counteractive manner (Awuh and Flo, 2017). As well as, *M. tuberculosis* generates and secretes antioxidant enzymes (e.g., superoxide dismutases and glutathione peroxidase), helping them to persist in an abnormal redox environment. Additionally, *M. tuberculosis* possesses the ability to repair and remove oxidative damaged proteins (Jaeger, 2007). Antioxidative system highly effective in protecting intracellular bacteria against ROS stress was discovered in other GI pathogens as well, it was shown that *Coxiella burnetii* produces an acid phosphatase with inhibitory effects on free radical release from phagocytes NADPH oxidase (Hill and Samuel, 2011), and *Francisella tularensis* synthesizes factors which inhibit or disrupt NADPH oxidase activity, conducing bacterial colonization and virulence (Mccaffrey et al., 2010).

#### Interference in Immunoresponses

In the context of an infection with GI pathogens, multiple cytokines (both pro- and anti-inflammatory cytokines) implicate in controlling or promoting pathogenesis, which are important for the fate of GI pathogens. It was shown that GI pathogens can manipulate cytokine responses to their advantage. For example, *Mycobacteria* increase secretion of IL-10, the anti-inflammatory cytokine by macrophage, maintaining the macrophages in a resting state (Mcclean and Tobin, 2016); induce the expression of SH2 domain-containing protein (CISH) and host Suppressor of Cytokine Signaling 1 (SOCS1), thus dampening pro-inflammatory responses (Duncan et al., 2017). Also, mycobacterial infection results in the expansion of regulatory cells. All these findings are helpful for understanding the biology of microbes associated with GI.

### Metabolic Regulation

For the intracellular bacteria, a suitable substance supply, including nutrients, ions and carbon influences their behavior and plays a crucial role in their persisting long enough within host cells. However, the substances needed for the metabolism of intracellular bacteria are found only in the infected host cells. Therefore for this purpose, intracellular bacteria reprogram their metabolism to contend with the limited metabolic resources of the host. They rely on various or similar host-derived carbon sources to replicate, e.g. *L. monocytogenes* residing within the host cell cytosol uses host glycerol and G6P as sources of carbon and energy (Chatterjee et al., 2006; Joseph et al., 2006), *Brucella* induces the upregulation of glucose uptake and glycolysis to support their growth and survival in the intracellular niche (Czyz et al., 2017). Some bacteria, e.g. *M. tuberculosis* acquire nutrients *via* multiple metabolic pathways (Bloch and Segal, 1956; Mehrotra et al., 2014).

Metal ions, including iron, copper, manganese and zinc, as components of metalloproteins or as structural elements for enzymes are required in many biological processes. During infection, the host restricts the availability of essential metals from invading pathogens. Meanwhile, the toxicity of metals such as zinc and copper can be used as a host defense mechanism to facilitate bacterial killing (Porcheron et al., 2013). Studies have shown that pathogens evolve sophisticated systems to control the transportation of these metals to ensure their physiological needs while countering metal toxicity, e.g. *M. tuberculosis* and *E. coli* are able to acquire soluble iron from host iron proteins through siderophores (Chakraborty et al., 2012; Liu et al., 2014), ferroportin expressed in the membrane of *M. tuberculosis*-containing phagosomes can provide intraphagosomal iron that favors the pathogen (Van Zandt et al., 2008); *S. typhimurium* induces the formation of estrogen-related receptor-γ, which triggers hepcidin expression and iron retention in macrophage cells (Kim et al., 2014). In contrast, *M. tuberculosis* synthesizes mycobacterial copper transport protein B (MctB), ATPase CtpV (Hodgkinson and Petris, 2012; Neyrolles et al., 2013) and p-type ATPase (Neyrolles et al., 2013), which enable them to resist metal intoxication within the phagosomes of macrophages.

In addition to the above strategies used in survival and persistence of GI pathogens, antimicrobial resistance (AMR) is also closely related to intracellular survival of the pathogen upon antimicrobial agents, which lead to treatment failure. Under extreme nutrient-limiting conditions or during latency, GI pathogens obtain additional survival advantages by slowing growth, reducing respiration rate, etc., increase their tolerance against stresses imposed upon them (Wayne and Hayes, 1996; Leistikow et al., 2010). Associated discoveries will not be included in this review.

## Predisposing Factors in Hosts

### Microbial Recognition Defect

PRRs of the host identify microbial pathogens and form the foundation of the innate immune system. PRRs include Toll-like receptors (TLRs), retinoic acid inducible gene-I(RIG-I)-like receptors (RLRs), C-type lectin receptors, oligo-adenylate synthetase (OAS)-like receptors, absent in melanoma 2 (AIM2)-like receptors and nucleotide-binding oligomerization (NOD)-like receptors (NLRs) (Thompson et al., 2011). Based on N-terminal domain, NLRs are divided into four subfamilies, NLRA, NLRB, NLRC, and NLRP, which have a common domain organization with a central NOD. Different bacterial molecules have been characterized as ligands or stimulators of PRRs (Takeuchi and Akira, 2010).

Defects in PRR signaling contribute to the pathogenesis of different GIs. Several single-nucleotide polymorphisms (SNPs) in *TLR1* have been recognized to be associated with susceptibility or resistance to leprosy and leprosy reactions (Johnson et al., 2007; Marques Cde et al., 2013). Genetic variants of *TLR1, 2, 4*, and *6* were shown to be involved in the activation of immune cells during the development of CD, sarcoidosis and leprae (Rock et al., 1998; Arbour et al., 2000; Ferguson et al., 2007; Chen et al., 2010; Marques Cde et al., 2013).

*NOD2* in the cytoplasm recognizes bacterial peptidoglycan in the cell walls of Gram-negative and Gram-positive bacteria, leads to NF-κB activation and production of IL-6, IL-1b, TNF-α, IL-8 and α-defensins (Negroni et al., 2018). Also it interacts with autophagy-related proteins to help destroy intracellular pathogens (Naser et al., 2012). *NOD2* was one of the first PPRs identified to be a strongly associated genetic risk factor for CD (Ogura et al., 2001). Likewise, polymorphisms in the *NOD2* gene region were found to be associated with leprosy reactions (Berrington et al., 2010). In addition, polymorphisms in the *NLRP1, NLRP3* and *NLRP6* were linked with CD and leprosy susceptibility as well (Villani et al., 2009; Pontillo et al., 2013).

### Autophagy Defect

Autophagy is an important intracellular process by which invading pathogens are degraded inside the lysosomes (Deretic et al., 2013). The defect in autophagy has been shown to result in excess production of cytokine (Van De Veerdonk and Dinarello, 2014), several autophagy-related genes have been identified that predispose individuals to a higher GI diseases risk. For example, *ATG16L1* is expressed in T cells, antigen-presenting cells and intestinal Paneth cells (Cadwell et al., 2008). It interacts with IRGM and NOD2 to form a molecular complex to regulate autophagy responses to microbial invading (Chauhan et al., 2015). It was shown that knocking down *ATG16L1* reduces the ability of cells to capture bacteria and abrogates autophagy of *S. typhimurium* in host cells, which may promote the onset of CD (Kuballa et al., 2008; Schultz et al., 2017). Mutations in autophagy-related gene *PTPN2* not only lead to defective autophagosome formation, but also promote T cell differentiation into Th1 and Th17 types (Spalinger et al., 2015; Spalinger et al., 2018). Two other autophagy-related genes, *LRRK2* and *MUC19* were also reported to be associated with CD risk (Barrett et al., 2008; Tong et al., 2010). *PRKN/PARK2* was identified as a genetic susceptibility factor for leprosy and BU(Table 2), and was shown to play a role in the degradation of intracellular *Salmonella, Mycobacteria* and *Listeria* (Manzanillo et al., 2013). CGD is an inherited GI disease caused by a defect in the production of reactive oxygen species, but both mouse studies and human studies have shown that defective autophagy is involved in its pathogenesis similarly (Van De Veerdonk and Dinarello, 2014), although the exact mechanisms are not yet clear.

### Oxidation Defect

NADPH oxidase, an enzyme mainly contained in the plasma membrane of macrophages and neutrophils, represents an important defense mechanism in microbial killing (Minakami and Sumimotoa, 2006). The functional NADPH oxidase complex is composed of 5 subunits. The genes encoding the five subunits of the NADPH oxidase enzyme are CYBA, CYBB, NCF1, NCF2 and NCF4. Molecular defects in any one of these genes can result in CGD, which is characterized by the impaired production of ROS and failure to eliminate pathogens and tissue granuloma formation (Idh et al., 2017).

Notably, nearly 50% of CGD patients develop an inflammatory bowel disease that resembles CD (Marks et al., 2009). Consistently, a recent study identified missense mutations in *CYBB, CYBA, NCF1, NCF2* and *NCF4* in some patients with CD (Denson et al., 2018). In (1990), Nielsen et al. reported that alveolar macrophages from patients with sarcoidosis showed a weak oxidative burst response *in vitro* stimulation, which is thought to be involved in the pathology of pulmonary sarcoidosis (Nielsen et al., 1990). However, a genetic polymorphism investigation failed to find a significant association of polymorphisms in *CYBB, CYBA, NCF1, NCF2, NCF4* that led to increased susceptibility to sarcoidosis (Lee et al., 2006). In (2017), Werner et al. generated a mouse model of GI using a strain of *P. acnes* isolated from a patient with sarcoidosis, and showed that a deficiency in *CYBB* is linked with increased granuloma formation in the lung (Werner et al., 2017).

In addition to *NADPH*, some gene coding enzymes related to oxide metabolism are also involved. For instance, *SOD2* encodes superoxide dismutase 2, a homotetrameric mitochondrial enzyme that converts superoxide derivatives of oxidative phosphorylation into hydrogen peroxide and diatomic oxygen (Wiener et al., 2007). A family-based analysis revealed that *SOD2* is a risk gene conferring susceptibility to leprosy (Ramos et al., 2016). With the development of gene research technology, more potential risk gene may be discovered in the future.

### Dysregulated Immunoresponses

Resistance to infection involves a set of interrelated defenses. If the recruited and activated macrophages are unable to remove invading pathogens efficiently, a further immune response may be triggered, which works to control the pathogen.

Antigen presentation is important for the initiation of adaptive immune responses. Due to the crucial role of human leukocyte antigens (HLAs) in antigen presentation and immunomodulation, the SNPs of major histocomatibility complex (MHC) locus have been investigated intensively, which enhances our understanding of the underlying mechanisms. Studies have shown that HLA alleles and haplotypes are involved genetic factors controlling susceptibility to GI diseases, including OFG (Gibson and Wray, 2000), CD (Orchard et al., 2002), leprosy (Jarduli et al., 2013), TB (Sveinbjornsson et al., 2016) and sarcoidosis (Design of a Case Control Etiologic Study of Sarcoidosis (ACCESS). ACCESS Research Group, 1999). Certain HLA alleles, e.g. HLA-DPB1*1701 allele and DPB1*2:01 allele have been recognized as risk factor for GI diseases in humans (Richeldi et al., 1993).

Outside SNPs of MHC, the genes associated with the development, proliferation, apoptosis, migration of immune cells, and antibody production may affect the clinical phenotype and behavior of GI disease (**Table 2**), Not only that, there is crosstalk between different genes, for instance, *NOD2* activation triggers autophagy of immune cells with the participation of *ATG16L1*, and deficiency in *ATG16L1* heightens cytokine production *via NOD* (Sorbara et al., 2013), the patients with high-risk *NOD2* or *ATG16L1* variants exhibit impaired MHCII antigen presentation (Cooney et al., 2010). The absence of effective crosstalk may lead to altered inflammation, increasing susceptibility to development of GIs further.

**Table 2:** Summary of candidate genes linked to GI diseases.

GI disease	Candidate genes	Potential mechanism of action	References
**TB**	*ASAP1*	Cytoskeleton remodeling	(Curtis et al., 2015)
	*CD209, TLR1,2,4,8,10*	Pathogen recognition	(Bulat-Kardum et al., 2015; Salie et al., 2015; Yi L. et al., 2015; Zhao et al., 2015; Jafari et al., 2016)
	*IRGM*	Autophagy	(Yuan et al., 2016)
	*AGMO, FOXP1*	Macrophage function	(Grant et al., 2016)
	*TOX, DUSP14, CCL2, CCL5, CCL13*	Monocyte and T cell function	(Thuong et al., 2008; Grant et al., 2013; Hijikata et al., 2016; Kouhpayeh et al., 2016; Nonghanphithak et al., 2016)
	*SP110, IFNA1, IFITM3, TYK2*	Interferon signaling	(Bhanothu et al., 2015; Naderi et al., 2016; Zhang et al., 2017; Boisson-Dupuis et al., 2018)
	*IL17A, IL17F, IL27, IL8*	Immunoregulation	(Braun et al., 2015; Du et al., 2015; Hu et al., 2020; Li et al., 2020)
	*CTSZ*	Protein localization and activity	(Adams et al., 2011)
	*HLA-DQA1, HLA-DRB1, MR1*	Guiding antigen specific T cell immune response	(Sveinbjornsson et al., 2016; Seshadri et al., 2017)
	*MAFB*	Regulation of lineage-specific hematopoiesis	(Mahasirimongkol et al., 2012)
	*TNF, NOTCH4*	Apoptosis, differentiation and proliferation	(Yi Y.X. et al., 2015; Zhang et al., 2020)
	*MC3R*	Regulation of energy homeostasis	(Adams et al., 2011)
	*UBE3A*,	Ubiquitin protein degradation	(Cervino et al., 2002)
	*NCF2, CYBA*	Oxidation	(Liu et al., 2016; Jiao et al., 2020)
	*CYP7A1, VDR, VDBP, ITPA*	Metabolism	(Qrafli et al., 2014; Lee et al., 2016; Nakauchi et al., 2016)
	*HSPEP1, UBLCP1, SIGLEC15*	Unknown	(Mahasirimongkol et al., 2012; Sobota et al., 2016; Bhattacharyya et al., 2019)
**leprosy**	*CUBN, NRAMP1/SLC11A1, VDR*	Vitamin and iron metabolism	(Hatta et al., 2010; Grant et al., 2014; Neela et al., 2015)
	*LACC1/C13orf31, SOD2*,	Cellular ROS production	(Ramos et al., 2016; Lahiri et al., 2017)
	*NOD2, TLR1, NLRP1*	Pathogen recognition	(Schuring et al., 2009; Zhang et al., 2009; Berrington et al., 2010; Marques Cde et al., 2013; Pontillo et al., 2013)
	*HLA-C, HLA-DQA1, HLA-DRB1, HLA-DQB1*	Guiding antigen specific T cell immune response	(Geluk and Ottenhoff, 2006; Alter et al., 2011; Jarduli et al., 2013; Zhang et al., 2019)
	*LTA, TNF, TNFSF15*	Apoptosis and inflammation	(Zhang et al., 2009; Cardoso et al., 2011)
	*IL23R, GATA3, IL10, RIPK2*	Immunoregulation	(Zhang et al., 2009; Zhang et al., 2011; Medeiros et al., 2016)
	*LRRK2/RIPK7, RAB32, PARK2/PRKN, MRC1*	Autophagy	(Mira et al., 2004; Zhang et al., 2009; Alter et al., 2010; Zhang et al., 2011)
	*NEBL*	Focal adhesion	(Grant et al., 2014)
	*PACRG*	Protein degradation	(Mira et al., 2004)
	*HIF1A*	Apoptosis and metabolism	(Wang et al., 2018)
	*TYK2*	Interferon signaling	(Liu et al., 2017)
	*CCDC122*	Unknown	(Zhang et al., 2009)
**CD**	*ATG16L1, IRGM, LRRK2, MUC19, PTPN2, PTPN22*	Capture bacteria, autophagy	(Barrett et al., 2008; Cadwell et al., 2010; Tong et al., 2010; Spalinger et al., 2018)
	*Annexin A11*	Apoptosis, proliferation	(Reischl et al., 2020)
	*LACC1/C13orf31, DUOX2, NOX2/CYBB, CYBA, NCF1, NCF2, NCF4*	Oxidation	(Cleynen et al., 2013; Denson et al., 2018)
	*HLA-A2,HLA-DR1, HLA-DQw5*	Guiding antigen specific T cell immune response	(Orchard et al., 2000; Orchard et al., 2002)
	*IL-23R, IL10, IL6, JAK2, STAT3, CCR6, ICOSLG, PUS10, SLC22A23*	Immunoregulation and iron metabolism	(Yamamoto et al., 2000; Petit-Bertron et al., 2005; Van Limbergen et al., 2009; Festen et al., 2011; Cleynen et al., 2013; Xu et al., 2015)
	*NOD2, NLRP3, NLRP6, CARD9, TLR1,2,4,6*,	Pathogen recognition	(Ogura et al., 2001; Van Limbergen et al., 2009; Villani et al., 2009; Kordjazy et al., 2018)
	*TNFSF15, TAB2, PRDM1*	Apoptosis, inflammation and immunoregulation	(Barrett et al., 2008; Cleynen et al., 2013)
	*C10orf6*	Unknown	(Franke et al., 2008)
**Sarcoidosis**	*Annexin A11, XAF1*	Apoptosis and proliferation	(Li et al., 2010; Levin et al., 2014)
	*BTNL2, NOTCH4, CCDC88B, ZNF592*	T cell response	(Valentonyte et al., 2005; Adrianto et al., 2012; Fischer et al., 2012; Lareau et al., 2015)
	*COX2, NOX2/CYBB*	Oxidation	(Christophi et al., 2014; Werner et al., 2017)
	*HLA-DRB1, DPB1, DQB1, TAP2*	Guiding antigen specific T cell immune response	(Design of a Case Control Etiologic Study of Sarcoidosis (ACCESS). ACCESS Research Group, 1999; Hulpke et al., 2012; Malkova et al., 2020)
	*IL23R, IL12B*	Immunoregulation	(Kim et al., 2011; Fischer et al., 2015)
	*NOD2, TLR2*,	Pathogen recognition	(Kanazawa et al., 2005; Chen et al., 2010)
	*SH2B3*	Negative regulator of TNF signaling	(Fischer et al., 2015)
	*RAB23*	Autophagy	(Davoudi et al., 2018)
	*TNF, LTA*	Apoptosis and inflammation	(Mcdougal et al., 2009; Song et al., 2014)
	*MAGI1*	Epithelial and endothelial cell-cell contacts	(Garman et al., 2020)
**BU**	*ATG16L1, PARK2/PRKN*	Autophagy	(Capela et al., 2016)
	*IFNG*	Interferon signaling	(Bibert et al., 2017)
	*NOS2*	Immunoregulation	(Bibert et al., 2017)
	*NOD2*	Pathogen recognition	(Capela et al., 2016)
	*NRAMP1/SLC11A1*	Iron metabolism	(Stienstra et al., 2006)
**CGD**	*NOX2/CYBB, CYBA, NCF1, NCF2, NCF4*	Oxidation	(Idh et al., 2017)
**OFG**	*HLA-A3, B7, DR2, HLA-B16, HLA-CW3*	Guiding antigen specific T cell immune response	(Gibson and Wray, 2000)
**GPA**	*SERPINA1*	Protein activity	(Lyons et al., 2012)
	*HLA-DOA, DP, DPA1, DPB1, DPB2, DQ, DRB1*	Guiding antigen specific T cell immune response	(Cao et al., 2011; Lyons et al., 2012; Xie et al., 2013)
	*TLR9*	Pathogen recognition	(Husmann et al., 2014)
	*PTPN22, CTLA4, FCGR3B, IL2RA MOSPD2*	Immune response and T cell function	(Jagiello et al., 2005; Willcocks et al., 2008; Carr et al., 2009; Lyons et al., 2012)
	*DCTD, GHSR, HSD17B8, LEPR, RING1, RXRB*	Metabolism	(Wieczorek et al., 2010; Xie et al., 2013)
	*COL11A2*	Extracellular matrix	(Lyons et al., 2012)
	*COBL, ARHGAP18, SEMA6A*	Cell shape and cytoskeleton	(Lyons et al., 2012; Xie et al., 2013)
	*CD226, PRTN3*	Cellular adhesion, cell-cell contacts	(Wieczorek et al., 2009; Lyons et al., 2012)
	*IRF5*	Interferon signaling	(Wieczorek et al., 2010)
	*CCDC86, WSCD1*	Unknown	(Xie et al., 2013)

TB, tuberculosis; CD, Crohn’s disease; BU, Buruli ulcer; CGD, chronic granulomatous disease; GPA, Wegener’s granulomatosis; OFG, orofacial granulomatosis; ROS, reactive oxygen species.

Interestingly, many polymorphisms are not disease specific, some unexpected overlap in genetic architecture between different GI diseases have been revealed (**Table 2**), for example, CD loci were also markedly enriched in genes involved in leprosy. Generally, in most cases a negligible number of single mutations have not been found that cause GI diseases, and many of the associated genes are thought to combine to produce a predisposition, although their exact contribution to GI disease have not been fully evaluated. In addition, some other genes were selected as positional and functional candidates for association studies, but their function is not yet known (Medeiros et al., 2016; Nakauchi et al., 2016).

### Epigenetic Modifications

Extensive research into genetic predispositions that increase the susceptibility to GIs was performed though, in some investigations researchers still failed to uncover specific functional genes that are associated with the susceptibility to GIs, thus epigenetics arouses increasing attention. Epigenetics refers to changes in the activity, expression or function of genes that are not mediated by DNA sequence, mechanisms of epigenetics include DNA methylation, histone modifications, and non-coding RNA (Kitazawa et al., 2022). Data showed that epigenetic modifications, resulting from interactions between the host and exposome potentiate host susceptibility to GIs. This notion is compelling given that epigenetic alternations have been linked to bacterial infectious diseases (Compare et al., 2011). Consistently, an increased risk of developing CD among people migrating from low- to high-incidence regions of CD provided important epidemiological information to support the pathogenic role of epigenetic changes (Benchimol et al., 2015).

Studies have demonstrated some key metabolites of the bacteria, such as mycobacterial lipoprotein (Pennini et al., 2006), mannosylated lipoarabinomannan (ManLam) and Eis protein (Kim et al., 2012)serve as regulators of the host cellular transcriptional machinery, participating in epigenetic processes associated with GI (Miro-Blanch and Yanes, 2019); a variety of short-chain fatty acids (SCFAs) produced by bacteria can epigenetically regulate the immune response (Olza et al., 2017); microRNA was shown to be participated in the immune response to some GI pathogens (Kalla et al., 2015); global methylation analysis of peripheral blood mononuclear cells (PBMCs) from TB patients revealed that DNA methylation signatures may regulate certain immune responses *in vivo* (Chen et al., 2017). These findings offer new insights into the pathogenesis of GI diseases.

### Other Cells Involved

Recently, eosinophils and platelets in GI diseases were gradually recognized (Requena and Fernandez-Figueras, 2007; Lugo-Villarino and Neyrolles, 2014; Furuta et al., 2019). They all contain major granule proteins and a large amount of cytokines and chemokines (Requena and Fernandez-Figueras, 2007; Deppermann and Kubes, 2016), but their potential role in GI diseases was rarely addressed.

After a long period of dominance by classic immune cells, somatic cells, such as epithelial cells and fibroblasts have been discovered to be additional actors in the maintenance of the immunological integrity of the human body. The epithelium acts as a barrier, separating the bacteria from the immune cells, altered physical epithelial barrier function, a thinner mucus layer have been identified as risk factors for GIs (Hui et al., 2011; Turpin et al., 2018).

GI occurs in all tissue sites, however varies considerably in their degree of complexity, physical size and organization. The structural composition plays a role as a primary host-defense mechanism for containing bacteria, also provides a shelter for pathogens, the cause of the specific microarchitecture and inflammatory status of GI deserves further study.

### Other Predisposing Conditions

In addition to affecting people with inherent factors, as stated above, GI diseases usually affect patients with other predisposing conditions, e.g., lactation, psychological stress, pregnancy, intercurrent infections, puberty, vaccination or various environmental stimuli (Kahawita and Lockwood, 2008). Immunocompromised individuals, including those with leukemia, lymphoma, diabetes and uncontrolled HIV infection, and patients who are taking immune-suppressing biologics prescribed for common immune-mediated diseases and cancers are at risk for GI diseases (Robson, 2000; Doherty et al., 2008; Agarwal, 2011), which makes the situation more complex and changeable.

## Conclusions

GI diseases present challenges to scientific inquiries and clinical managements. The existing literature suggests that the unstable balance between bacterial virulence and host immunity determines the pathological features of the infections related to these diseases, however, the interconnecting mechanisms still remain largely elusive. Recently, microbial dysbiosis in the commensal community has received great attention in GI research, especially for the resident bacteria associated GIs. Taking advantage of high-throughput data on genetic and microbiome may open up a new avenue for GI research.

## Author Contributions

XW: Writing-Original draft preparation. YL: Conceptualization, supervision and validation. All authors contributed to the article and approved the submitted version.

## Funding

This work is supported by grants from the National Clinical Research Center for Oral Disease of China (LCA202008) and the State Key Laboratory of Military Stomatology (2018ZB01).

## Conflict of Interest

The authors declare that the research was conducted in the absence of any commercial or financial relationships that could be construed as a potential conflict of interest.

## Publisher’s Note

All claims expressed in this article are solely those of the authors and do not necessarily represent those of their affiliated organizations, or those of the publisher, the editors and the reviewers. Any product that may be evaluated in this article, or claim that may be made by its manufacturer, is not guaranteed or endorsed by the publisher.

## Abbreviations

GI, granulomatous inflammation; TB, tuberculosis; CD, Crohn’s disease; BU, Buruli ulcer; GPA, granulomatosis with polyangiitis; OFG, orofacial granulomatosis; CGD, chronic granulomatous disease; IB, intracellular bacteria; NADPH, transmembrane nicotinamide adenine dinucleotide oxidase; AMR, antimicrobial resistance; PRRs, pattern recognition receptors; TLRs, Toll-like receptors; NKs, natural killer cells; DCs, dendritic cells; ROS, Reactive oxygen species; SNPs, single-nucleotide polymorphisms; NLRs, nucleotide-binding oligomerization (NOD)-like receptors; HLAs, human leukocyte antigens; MHC, major histocomatibility complex.
